# Understanding the relationships between dietary risk behaviour and social factors in older adults

**DOI:** 10.1093/eurpub/ckac130.106

**Published:** 2022-10-25

**Authors:** C Geigl, L Spagert, J Loss, M Leitzmann, C Janssen

**Affiliations:** 1 Department of Applied Social Sciences, Munich University of Applied Sciences, Munich, Germany; 2 Department of Epidemiology and Preventive Medicine, University of Regensburg, Regensburg, Germany; 3 Department of Engineering and Management, Munich University of Applied Sciences, Munich, Germany; 4 Department of Epidemiology and Health Monitoring, Robert Koch Institute, Berlin, Germany

## Abstract

**Background:**

The relationships between social factors and dietary risk behaviour in older adults have not yet been thoroughly investigated. In this analysis, we aimed to develop a brief index of dietary risk behaviour and examine its associations with sociodemographic, socioeconomic, psychosocial, and behavioural factors.

**Methods:**

A community-based postal survey was conducted to collect cross-sectional data from German adults aged 65 and older (n = 1687; 33% response proportion; 52% female). Using principal component analysis, we developed a 3-item dietary risk behaviour index (DRB), including the food groups vegetables/fruit, whole grains, and dairy products. Dietary risk behaviour was defined as food group consumption frequencies below national dietary recommendations. Multiple linear regression was used to analyse associations between dietary risk behaviour and social factors.

**Results:**

Physical activity, female gender, education level, and social support were negatively associated with dietary risk behaviour, while alcohol consumption and smoking were positively associated (Adj. R2 = 0.16, p < 0.001). The brief DRB based on vegetables/fruit, whole grains, and dairy products has proven to be appropriate in analysing dietary behaviour among older adults.

**Conclusions:**

A better understanding of the relationships between social factors and dietary risk behaviour among older adults can assist in group-specific targeting of dietary-related interventions. Demand-oriented dietary interventions should address underlying social factors to reduce inequities in dietary risk behaviour among older adults. The results may be transferable to municipalities in high-income European countries.

**Key messages:**

- Dietary risk behaviour among older German adults is associated with sociodemographic, socioeconomic, psychosocial, and behavioural factors.
- Social conditions should be considered when targeting group-specific interventions to reduce socially determined inequities in dietary risk behaviour among older adults.

